# Multiple endocrine neoplasia 2: an overview

**DOI:** 10.1177/20406223221079246

**Published:** 2022-02-25

**Authors:** B Saravana-Bawan, JD Pasternak

**Affiliations:** University Health Network, University of Toronto, Toronto, ON, Canada; Section Head, Endocrine Surgery and Oncology, Division of General Surgery, Sprott Department of Surgery, Toronto General Hospital, University Health Network, 200 Elizabeth Street, Toronto, ON M5G 2C4, Canada

**Keywords:** hyperparathyroidism, medullary thyroid cancer, MEN2, multiple endocrine neoplasia, pheochromocytoma

## Abstract

This review article discusses the diagnosis and treatment of patients with multiple endocrine neoplasia type 2 (MEN2). The most common tumors associated with MEN2 are those of the parathyroid, thyroid, and adrenal glands. Additional manifestations include characteristic clinical phenotypes or features as described in the article. This review provides an overview of clinical manifestations, screening, diagnosis, treatment, and surveillance of patients with MEN2.

## Introduction

Multiple endocrine neoplasia type 2 (MEN2) is an autosomal dominant familial disorder causing tumors within various endocrine glands. Classically, the disease was characterized by two distinct phenotypes: MEN2A and MEN2B; however, more recently, disease characteristics have been linked to its exact pathogenic variant within the rearranged during transfection (*RET*) gene.^1^ The *RET* proto-oncogene is located on chromosome 10q11.2 and encodes a tyrosine kinase receptor which is primarily expressed in neuroendocrine and neural cells.^2^ This receptor has a crucial role in cell growth and differentiation.^3^

MEN2 is characterized by activating or ‘gain in function’ pathogenic variants *RET* proto-oncogene. While there are many associated tumors found in these patients, the classic entity is the development of medullary thyroid cancer (MTC), a rare thyroid malignancy of neuroectodermal origin. There is a spectrum of possible pathogenic variants in the *RET* proto-oncogene, either familial or sporadic in nature, which results in a spectrum of disease both in location and severity.^4,5^

MEN2 is a relatively rare disease with a prevalence of 1–10 per 100,000.^6^ Of the subtypes of MEN2, the most common is MEN2A, accounting for over 80% of cases of MEN2.^4^ Of MEN2A cases, the most common pathogenic variant observed is cysteine amino acid substitutions at codon 634.^7^

In 1961, Dr. John H. Sipple first noted the occurrence of thyroid cancer, parathyroid adenoma, and pheochromocytoma in a patient he saw and since then the disease describing this phenotype, MEN2A, has been referred to as Sipple’s syndrome.^8^ This entity is characterized by MTC, primary hyperparathyroidism (PHPT), and pheochromocytoma. Over 90% of patients develop MTC, with up to 50% of patients developing pheochromocytomas and 30% developing PHPT.^9,10^ The likelihood of developing pheochromocytomas or PHPT is dependent on the *RET* proto-oncogene pathogenic variant.^11^ The majority of MEN2A pathogenic variants are inherited in an autosomal dominant pattern and are located in the *RET* proto-oncogene’s extracellular cysteine-rich domain of exon 10 (codons 609, 611, 618, or 620) or exon 11 (codon 634) with codon 634 pathogenic variants accounting for at least 50% of cases.^9,12,13^ There is a specific subset of MEN2A, familial medullary thyroid carcinoma (FMTC), which is characterized by the development of MTC alone and often related to pathogenic variants in non-cysteine rich domains.

MEN2B is the most aggressive variant of the MEN2 subtypes. Over 95% of MEN2B cases result from a pathogenic variant in codon 918 at exon 16 of the *RET* proto-oncogene.^9^ In contrast to MEN2A, MEN2B cases are more often the result of *de novo* germline pathogenic variants, which often leads to delayed diagnosis.^14,15^ This syndrome is characterized by the development of MTC in all patients, pheochromocytomas in up to 50% of patients, and a characteristic clinical phenotype of a marfanoid body habitus and neuromas.^11^

Recognition of MEN2 syndromes is key, as early diagnosis and genetic testing allow for screening and preventive surgery which results in a significant reduction in associated morbidity and mortality. The aim of this review is to provide a summary of tumors associated with MEN2 to aid in early recognition and management.

## Medullary thyroid carcinoma

MTC is a rare thyroid malignancy originating from the parafollicular C-cells, a small volume of neuroectodermal originating cells found within the thyroid follicular tissue mass. Familial MTC is preceded by the development of C-cell hyperplasia with subsequent MTC progression. C-cells produce calcitonin and accordingly, elevated calcitonin levels can be present in the disease course and used as a measure of disease progression after ablative surgery. Levels of carcinoembryonic antigen (CEA) can also be elevated in MTC and used as a tumor marker in surveillance.^16^ As MTCs arise from C-cells which do not take up iodine, it is not classified as a differentiated form of thyroid cancer and is not responsive to radioactive iodine adjuvant treatment (RAI). MTC is also chemotherapy insensitive and accordingly, management is primarily surgical in nature.^2^ Fortunately, new targeted therapies such as tyrosine kinase inhibitors have shown promise in advanced and progressive MTC disease.

Overall, while MTC accounts for only 5% of all thyroid cancer cases, it makes up 15% of all thyroid cancer–related mortality due to its aggressive nature and increased likelihood of lymphatic and hematogenous spread.^17^ Metastasis initially occurs to cervical and mediastinal lymph nodes with advanced disease spreading to the lungs, liver, and bones.^16^ MEN2 accounts for 20% of all MTC, but MTC in this population is more aggressive, with bilateral and multifocal disease, and occurs earlier in life in comparison with sporadic MTC. Because of this increased risk of morbidity and mortality, it is recommended that all patients with MTC be screened for pathogenic variants in the *RET* proto-oncogene.^18^

The American Thyroid Association (ATA) has stratified patients with MEN2 from low- to high-risk groups based on the identified pathogenic variant in *RET* proto-oncogene and the severity of the associated clinical course of MTC. As established by the 2015 ATA guidelines, the highest risk pathogenic variant includes MEN2B patients with *RET* codon p.Met918Thr pathogenic variant. High-risk MEN2 pathogenic variants are *RET* codon p.Cys634 and p.Ala883Phe pathogenic variants. Moderate risk pathogenic variants include identified *RET* codon pathogenic variants other than those noted to be high or highest risk such as p.Cys609, p.Cys611, p.Cys618, and p.Cys620.^19^ *RET* pathogenic variants and outcomes are further outlined in Table 1.

**Table 1.** *RET* pathogenic variants and MTC outcomes.

ATA risk level	Exon	*RET* pathogenic variant
Moderate	8	p.Gly533Cys
	10	p.Cys609Phe/Gly/Arg/Ser/Tyr, p.Cys611Phe/Gly/Ser/Tyr/Trp, p.Cys618Phe/Arg/Ser
	11	p.Cys630Arg/Tyr, p.Asp631Tyr, p.Lys666Glu
	13	p.Glu768Asp, p.Leu790Phe
	14	p.Val804Leu/Met
	15	p.Ser891Ala
	16	p.Arg912Pro
High	11	p.Cys634Phe/Gly/Arg/Ser/Trp/Tyr
	15	p.Ala883Phe
Highest	16	p.Met918Thr

ATA, American Thyroid Association.

The prevalence of pathogenic variants varies by geographic and ethnic distribution. Studies have highlighted the varying prevalence of different genotypes and phenotypes which must be considered by clinicians if targeted testing is being employed. While the codon 634 in exon 11 are the most prevalent pathogenic variants for MEN2A, regional predominance can vary due to founder effect and common ancestry in different regions.^20^ In China, the most frequent pathogenic variant is localized to codon 634 in exon 11.^21^ In Italy, the genetic variations are more diverse with common exon mutations being identified at exon 11, 14, and 15. The exon 15 pathogenic variant is clustered predominantly in the north of the country. Within Denmark and Portugal the p.Cys611Tyr in exon 10 pathogenic variant, which rarely arises *de novo*, has a relatively high prevalence.^22,23^ In Spain the pathogenic variant of p.Cys634Tyr in exon 11 is predominant; whereas in Germany, variants within codon 790 and 791 in exon 13 have been identified as more common.^23^ In Greece. the most prevalent pathogenic variant among the familial cases of MTC is p.Gly533Cys in exon 8.^20^ Interestingly, the pathogenic variant of p.Cys618Arg in exon 10 is mostly found in neighboring Cyprus.^24^

There is complexity of management and risk stratification for these patients as several *RET* pathogenic variants of unknown significance exist. In these patients, risk of MTC and penetrance of disease are not well established. This uncertainty leads to difficult clinical decision making and the benefits of surveillance compared to prophylactic surgical intervention must be carefully considered by the clinicians, patients, and their families.^25^ It should also be noted that evidence suggests that the ATA risk categories for MTC apply to clinical onset and progression of disease but do not relate to survival outcomes once MTC is clinically present. Once MTC has developed, cure rate and survival rates seem to depend on the disease stage rather than the pathogenic variant.^26^

Once a *RET* pathogenic variant of known significance is identified, the clinical team may recommend upfront intervention or surveillance. As the age of onset of MTC and penetrance of MTC in MEN2 varies by subtype, prophylactic thyroidectomy may be an initial treatment.^2,15^ For patients with the highest risk pathogenic variants, prophylactic thyroidectomy is recommended by age 1, and by age 5 for other high-risk patients. For those classified as moderate risk, a decision can be made for surveillance with cervical neck ultrasounds and calcitonin levels annually with deferral of surgery until clinical disease is evident. These children can be offered prophylactic thyroidectomy after age 5. Decision on the timing of surgery can be made in context of preference and availability for serial screening and based on pathogenic variant specific penetrance and age of anticipated MTC onset.^15^ Given the high morbidity of thyroidectomy in young patients and the lack of availability of high-volume pediatric thyroid surgeons, monitoring is often chosen by practitioners. Early intervention is critical as delayed diagnosis and treatment results in metastatic disease at time of diagnosis, significantly affecting outcomes including morbidity.^27^ Studies demonstrate that patients with hereditary MTC who are diagnosed and treated with screening have survival similar to that of the general population. This is contrasted with worse survival in patients with regional or distant metastasis. This highlights the importance of screening and intervention prior to the development of advanced disease.^28^

Although MEN2 is often associated with a germline *RET* pathogenic variant, up to 50%, of cases arise *de novo*.^27^ Accordingly, these patients who lack a family history are not screened, leading to diagnosis later in life. Unfortunately, for these patients, diagnosis is often made in the setting of metastatic disease leading to significantly worse outcomes.^29^

Evidence of local invasion or lymph node metastasis is the main determinant for the extent of surgery. Patients with early disease should undergo total thyroidectomy with prophylactic central neck dissection to remove lymph nodes which may harbor subclinical disease.^18^ Before treatment, we suggest patients have cross-sectional imaging of the neck and chest to determine the extent of disease especially in cases where calcitonin levels are elevated. Marked elevations in serum calcitonin, particularly over 500 pg/ml may suggest metastatic disease outside of the neck, particularly in the lungs or liver. There is no consensus on the indications for prophylactic lateral neck dissection. The presence of lateral neck disease however does necessitate therapeutic lateral neck dissection of levels II, III, IV, and V on the ipsilateral side. Patients with metastatic disease may benefit from less aggressive resections based on disease extent for palliation.^6,15,18^ Due to the association of inherited MTC with pheochromocytomas and PHPT, all patients should undergo testing with plasma metanephrines and subsequent abdominal cross-sectional imaging to ensure safe anesthetic. We also suggest serum calcium and PTH to determine whether concurrent hyperparathyroidism exists prior to thyroidectomy.

MEN2A has the most associated *RET* pathogenic variants and therefore the broadest spectrum of clinical disease. For this reason, Oncologists have shifted to classifying patients based on *RET* pathogenic variant rather than MEN classification which better describes clinical manifestations but imprecisely describes penetrance, outcomes, and thereby treatment and surveillance requirements.

FMTC is a variant of MEN2A, characterized by MTC without or decreased penetrance for other associated endocrinopathies such as pheochromocytoma or PHPT. Pathogenic variants of *RET* proto-oncogene associated with low risk of other endocrinopathies but persistent risk of MTC include pathogenic variants of codons 609, 611, 630, 768, 790, 804, and 891.^15^ FMTC is the least aggressive variant of inherited MTC. Disease onset is usually from the third to the fifth decade of life.^3,17^

MEN2B is characterized by the more aggressive forms of MTC, with higher risk pathogenic variants of ATA risk level and disease onset in the first decade of life. Reports of malignancy shortly after birth, notably with M918T pathogenic variants cause treatment difficulties in many health settings. The course of MTC in this population is primarily responsible for increased mortality in patients with MEN2B, although it is unclear if advanced tumor stage at presentation or more aggressive pathology is the key factor.^16,30^ Accordingly, prophylactic thyroidectomy is strongly recommended within the first year of life without delay. Prior to the implementation of standard prophylactic thyroidectomy, the mean age of death for patients with MEN2B was 21.^31^ In contrast, patients who receive prophylactic thyroidectomy have almost 100% 10-year survival.^32^ The consequences of surgery must always be considered in decision making, with higher rates of complications noted for pediatric in comparison to adult thyroidectomy. Long-term complications include recurrent laryngeal nerve injury and permanent hypoparathyroidism, the latter having double the rates compared to those in adult surgical patients (5% *versus* 2%).^33,34^ The risks of surgery do increase with disease burden, highlighting the importance of observant screening in patients whose pathogenic variant risk does not warrant prophylactic thyroidectomy and elect for screening.^35^

Survival for patients with MEN2 related MTC treated surgically is dependent upon disease stage and burden. Stage-dependent outcomes appear similar between patients with both sporadic and hereditary MTC.^36,37^ Post-resection, all patients with MEN2 should be followed with surveillance for recurrent disease. In addition, any patients with MTC, or a constellation of two MEN2 tumors, should undergo *RET* genetic testing and children of MEN2 patients should have genetic testing performed within the first 6 months of life.^18^ The most common sites of *RET* pathogenic variants are on exon 5, 10, 11, 13, 14, 15, and 16 and these should be assessed with genetic testing.^19^ Surveillance should consist of baseline calcitonin and CEA levels, 3 months post resection and then continued every 6 months for a year and then annually. Cervical ultrasound should be performed 6 months after resection and then again as indicated by elevated calcitonin levels. C-cell production of calcitonin and subsequent concentration levels are directly related to C-cell mass making it a useful tumor marker for MTC. Calcitonin can be used to assess initial disease burden and followed post resection to assess disease progression in patients with metastatic MTC. A serum calcitonin level over 500 pg/ml is associated with the presence of metastatic disease and should prompt more extensive staging workup with a CT neck, hepatic phase CT or MRI, axial MRI, and bone scintigraphy. Discordant calcitonin levels compared to structural disease burden can also be concerning. Low or normal levels of calcitonin in the setting of metastatic disease is a poor prognostic marker which may indicate dedifferentiation into poorly differentiated disease, an entity characteristically more aggressive and associated with worse outcomes.^19^

Treatment of metastatic MTC depends on disease burden. Patients with persistent disease or locoregional recurrence within the neck can be treated with compartment-based neck dissection. Distant metastatic disease is usually not curable but treatment options to control disease and provide symptom relief are available. To understand a specific patient disease course, calcitonin doubling time represents the rate of tumor burden over a period of time. For patients with calcitonin doubling times over 2 years, usually representing stable disease, initiation of systemic treatment may not be recommended in favor of close surveillance.^38,39^

There are no widely used radiolabeled molecules or cytotoxic chemotherapeutic options for MTC. First-line systemic treatment consists of tyrosine kinase inhibitors (TKIs) which target and inhibit *RET* kinase and vascular endothelial growth factor receptor (VEGFR) and epidermal growth factor receptor (EGFR) signaling and are indicated in metastatic patients with progressive disease or those with high disease burden.^40–44^ Multitargeted kinase inhibitors such as vandetanib are approved for the treatment of MTC.^45^ The response of these multitargeted kinase inhibitors for MTC is varied from 12% to 65% and utilization can be limited by adverse effects mainly thought to be due to simultaneous inhibition of non-*RET* kinases such as VEGF. Accordingly, the current research assessing the utilization of selective *RET* inhibitors, such as selpercatinib, in the treatment of *RET*-altered malignancies such as MTC demonstrates the efficacy and reduced adverse effects in initial studies.^45^

Specific sites of metastatic disease may be considered for treatment, especially in settings of oligometastatic disease. Isolated brain metastases can be offered surgical resection or external beam radiation therapy (EBRT). Bone metastasis causing fracture or with likely progression to fracture should be treated surgically, with ablation or cement injection. Spinal compression requires treatment with urgent surgical decompression. Other bone metastases which are symptomatic should be treated with denosumab, bisphosphonates, or EBRT. Solitary lung metastasis can be considered for surgical resection or local therapies dependent on size and location. Isolated hepatic metastasis can be considered for surgical resection or chemoembolization.^19^ Multidisciplinary collaboration is paramount to determine the best treatments for these complex patients.

## PHPT

PHPT is present in 30% of patients with MEN2A and is not in those with MEN2B or FMTC.^2,18^ Similar to other neoplastic entities of MEN2A, more aggressive pathogenic variants will cause PHPT to manifest at a younger age. Pathogenic variants of codon 634 are most commonly associated with MEN2A-related PHPT.^46^ The phenotype of PHPT can vary with a given pathogenic variant due to the differences in penetrance which is different than the more predictable rates of MTC in patients with MEN2A pathogenic variants.^47^ PHPT is very rarely the initial presentation for MEN2A. It generally is found later, in the fourth or fifth decade of life, although it has been diagnosed in select patients with codon 634 pathogenic variants as early as 5 years of age.^18,48^ Screening of patients with MEN2A for PHPT should begin at age 8 for those with aggressive pathogenic variants of codon 630 and 634 and at age 20 for those with other pathogenic variants. Screening includes annual PTH and calcium levels.^18^ Patients with MEN2A and PHPT are rarely symptomatic. If present, symptoms are identical to those observed in sporadic PHPT, such as nephrolithiasis osteoporosis and fatigue.^10^

Parathyroid disease in patients with MEN2 is clinically different than MEN1 as the disease is milder and mostly causes single adenomas compared to multigland progressive hyperplastic disease in the latter. Furthermore, many patients with MEN2A have had thyroidectomy and neck dissection for MTC and there is a high likelihood of incidental parathyroidectomy after neck dissection thereby potentially falsely decreasing the incidence of observed parathyroid disease. The incidence of incidental parathyroidectomy during total thyroidectomy is 16.2% with malignancy and neck dissection bother being independent risk factors for increased incidence.^49^ In fact, many patients with MEN2 after surgical treatment of thyroid cancer develop hypoparathyroidism. High-volume surgeons are best positioned to address the complex endocrine surgical disease of these patients.

## Pheochromocytomas

Pheochromocytomas are present in up to 50% of MEN2A and 2B patients. Pheochromocytomas associated with MEN2 tend to have a higher incidence of bilateral (synchronous or metachronous), multifocal and extra-adrenal disease.^18^ Pheochromocytomas typically present in the second to fourth decades of life in patients with MEN2. This is at least 10–20 years earlier than the age of presentation in patients with sporadic tumors.^16^ The most commonly identified *RET* proto-oncogene codon pathogenic variants are codon 634 and 918 and within these kindreds, bilateral disease often present.^2,10^

Symptoms are similar to those of sporadic disease with hypertension, palpitations, headaches, and diaphoresis being common.^50^ It has been noted that pheochromocytomas in patients with MEN2 have a tendency to sec*RET*e higher amounts of epinephrine than norepinephrine and accordingly, this patient population is more prone particularly to palpitations, anxiety, and paroxysmal hypertension.^51^

Pheochromocytomas are rarely the presenting disease of MEN2. However, they must always be considered and ruled out prior to the treatment of any associated conditions as well as those planning a pregnancy due to the risk of hypertensive crisis. If a pheochromocytoma is identified, it should be addressed as a priority. Screening should be performed for patients with MEN2 with plasma metanephrines or 24-h urinary catecholamines if the former is not available given higher sensitivity. Elevated biochemical testing necessitates further workup with cross-sectional adrenal imaging. The authors do not recommend functional imaging (Iodine 123 MIBG or the preferred Gallium 68 Dotatate PET scan) for diagnostic purposes. The majority of tumors in MEN2 are found within the adrenal, whereas extra-adrenal paragangliomas account for less than 10%.^52^ Initiation of annual screening should start at age 20 in patients with MEN2A and age 8 in patients with MEN2B specifically those with *RET* 918 pathogenic variant.^18^

Surgery for pheochromocytomas requires pre-operative optimization with alpha blockade and potential subsequent beta blockade for tachycardia due to the risk of hypertensive crisis. The surgical approach has changed considerably in recent times. Bilateral adrenalectomy was considered a classic standard given the risk of recurrent disease. Given the high morbidity of living without adrenal tissue, this approach evolved to unilateral adrenal resection for the involved gland, recognizing that up to 50% of patients with MEN2 did not develop bilateral disease in follow up.^53^ To avoid difficulties of living with full steroid hormone replacement, management of pheochromocytomas in patients with MEN2 has shifted toward staged and cortical-sparing or partial adrenal-sparing resection.^18^ The potential benefit of partial adrenalectomy must be weighed by the high risk of recurrent disease given the high likelihood of leaving medullary tissue behind with the normal cortical tissue. Some studies in high-volume centers have shown a low risk of recurrence noted to be 3–13% after adrenal resection.^54–56^ The authors would suggest only considering cortical-sparing adrenalectomy after previous unilateral adrenal resection, in less aggressive MEN2 variants and in a high-volume endocrine oncology center.

## Mucosal neuromas and other tumors

Patients with MEN2B have a set of features which creates a characteristic appearance including marfanoid habitus, narrow facies, pes cavus, pectus excavatum, scoliosis, mucosal neuromas, thickened, and everted eyelids with other mesodermal abnormalities.^50^

Additional features of patients with MEN2B which are not physically apparent include medullated corneal nerve fibers, ganglioneuromatosis.^50^ Mucosal neuromas are observed in almost 100% of patients with MEN2B. Once again, there is no predilection toward these tumors in patients with MEN2A or FMTC.^18^ These neuromas are found in any organs which have a submucosa ranging from the entire gastrointestinal tract to bronchi and urinary system with the gut being the most common site of manifestation.^16^ Symptomology related to mucosal neuromas can range from cosmetic with oral neuromas to more significant pathology with ganglioneuromatosis of the gastrointestinal tract having the potential to cause constipation, diarrhea, and even a pseudo-Hirschsprung’s disease with megacolon.^16,57^

Manifestations of MEN2 outside of the thyroid, parathyroid, and adrenal glands can be traced back to their *RET* proto-oncogene pathogenic variants. Table 2 includes a list of identified *RET* codon pathogenic variants associated with manifestations of MEN2 other than thyroid, parathyroid, and adrenal disease. This list represents a summary of what has been identified in the literature to date, note that the penetrance of manifestations is variable with some associations being noted in a few case series or studies. Regardless, awareness of associated non-classical features or diseases of MEN2 can initiate further testing resulting in potentially lifesaving detection and intervention in proband patients which is why these other manifestations are highlighted.

**Table 2.31,58 –60:** List of identified *RET* codon pathogenic variants associated with manifestations of MEN2.

Manifestation	Exon	*RET* Codon
CLA	11	634
	14	804*
Corneal nerve thickening	15	804*
	16	918*
HD	10	609, 611, 618, 620
Intestinal ganglioneuromatosis	15	883
	16	918
Marfanoid habitus	16	918
Renal agenesis	10	618*, 620*
	11	634*

CLA, cutaneous lichen amyloidosis; HD, Hirschsprung disease.

* Noted in case reports or series.

## Conclusion

MEN2 is a variable and complex disease syndrome with a spectrum of disease severity and features. Regardless of the identified pathogenic variant and known or unknown penetrance and phenotype, the key to management is close monitoring and early intervention. Due to the complexity of the disease, it is best managed through multidisciplinary care teams with the inclusion of genetic counselors, endocrine oncology, endocrine surgeons, radiologists, and primary care physicians.
